# Effects of induction of labour versus expectant management in women with impending post-term pregnancies: the 41 week – 42 week dilemma

**DOI:** 10.1186/1471-2393-14-350

**Published:** 2014-10-23

**Authors:** Joep C Kortekaas, Aafke Bruinsma, Judit KJ Keulen, Jeroen van Dillen, Martijn A Oudijk, Joost J Zwart, Jannet JH Bakker, Dokie de Bont, Marianne Nieuwenhuijze, Pien M Offerhaus, Anton H van Kaam, Frank Vandenbussche, Ben Willem J Mol, Esteriek de Miranda

**Affiliations:** Department of Obstetrics & Gynaecology, Radboud University Medical Center, Nijmegen, the Netherlands; Department of Obstetrics and Gynaecology, Academic Medical Center, University of Amsterdam, Amsterdam, the Netherlands; Department of Obstetrics and Gynaecology, Rijnstate Hospital, Arnhem, the Netherlands; Department of Obstetrics and Gynaecology, University Medical Center, Utrecht, the Netherlands; Department of Obstetrics and Gynaecology, Deventer Hospital, Deventer, the Netherlands; Midwifery practice ‘het Verloskundig Huys’, Zwolle, the Netherlands; Research Center for Midwifery Science, Faculty Midwifery Education & Studies Maastricht, ZUYD University, Heerlen, the Netherlands; KNOV (Royal Dutch Organisation for Midwives), Utrecht, the Netherlands; Department of Neonatology, Emma Children’s Hospital, Academic Medical Center, University of Amsterdam, Amsterdam, the Netherlands; The Robinson Institute, School of Paediatrics and Reproductive Health, University of Adelaide, Adelaide, 5000 SA Australia

**Keywords:** Pregnancy prolonged, Pregnancy post-term, Labour induced, Expectant management, Perinatal outcome, neonatal outcome, Maternal outcome, Maternal preferences

## Abstract

**Background:**

Post-term pregnancy, a pregnancy exceeding 294 days or 42 completed weeks, is associated with increased perinatal morbidity and mortality and is considered a high-risk condition which requires specialist surveillance and induction of labour. However, there is uncertainty on the policy concerning the timing of induction for post-term pregnancy or impending post-term pregnancy, leading to practice variation between caregivers. Previous studies on induction at or beyond 41 weeks versus expectant management showed different results on perinatal outcome though conclusions in meta-analyses show a preference for induction at 41 weeks. However, interpretation of the results is hampered by the limited sample size of most trials and the heterogeneity in design. Most control groups had a policy of awaiting spontaneous onset of labour that went far beyond 42 weeks, which does not reflect usual care in The Netherlands where induction of labour at 42 weeks is the regular policy. Thus leaving the question unanswered if induction at 41 weeks results in better perinatal outcomes than expectant management until 42 weeks.

**Methods/design:**

In this study we compare a policy of labour induction at 41 + 0/+1 weeks with a policy of expectant management until 42 weeks in obstetrical low risk women without contra-indications for expectant management until 42 weeks and a singleton pregnancy in cephalic position. We will perform a multicenter randomised controlled clinical trial. Our primary outcome will be a composite outcome of perinatal mortality and neonatal morbidity. Secondary outcomes will be maternal outcomes as mode of delivery (operative vaginal delivery and Caesarean section), need for analgesia and postpartum haemorrhage (≥1000 ml). Maternal preferences, satisfaction, wellbeing, pain and anxiety will be assessed alongside the trial.

**Discussion:**

This study will provide evidence for the management of pregnant women reaching a gestational age of 41 weeks.

**Trial registration:**

Dutch Trial Register (Nederlands Trial Register): NTR3431. Registered: 14 May 2012.

## Background

Post-term or prolonged pregnancy, defined as a pregnancy extended to or beyond 42 + 0 weeks or ≥ 294 days after the first day of last menstrual period, is associated with increased perinatal morbidity and mortality [1–10]. Therefore post-term pregnancy is considered as a high-risk condition which requires specialist surveillance and induction of labour at some stage, mainly because of a relative small group of undetected growth-restricted foetuses that is at risk for adverse perinatal outcome [11, 12].

In this era, with gestational age based on first trimester ultrasound, the incidence of post-term pregnancy is reduced to 3-5% or even less [13–15]. The risk on adverse perinatal outcome is considered to increase gradually rather than a steep increase from 42 weeks onwards, though the literature on this subject is ambiguous [16–18]. The Cochrane review on induction of labour for improving birth outcomes showed that a policy of labour induction at or beyond 41 completed weeks is associated with significant fewer perinatal deaths (22 trials, 9383 participants, RR 0.31 [95% CI 0.12-0.88]) although the absolute risk of perinatal death is small [18]. According to this review, labour induction doesn’t increase the risk of Caesarean section in women with a gestational age of 41 or 42 completed weeks [18]. However, only a few of the included studies had a policy of labour induction at 41 weeks in the intervention group. In other studies it is unclear whether labour was always induced at 41 weeks. Furthermore in most trials expectant management in the control groups continued far beyond 42 weeks [17–20]. In addition, recent observational studies showed that elective induction leads to similar increased maternal and foetal risks as induction on medical indication, in comparison to spontaneous onset of labour [21]. Recent meta-analysis showed that induction of labour on maternal or foetal indication in women with intact membranes reduces the risk of Caesarean section, thus leaving the question unanswered if induction at 41 weeks in obstetrical low risk women gives better perinatal outcomes and maternal outcomes than expectant management until 42 weeks [19, 22].

Because of the uncertainty regarding the management of (impending) post-term pregnancy, there is no consensus on the optimal timing of induction, leading to practice variation. Policy concerning low risk pregnancies at or beyond 41 weeks in the Netherlands varies from expectant management until 42 weeks, without extra surveillance, to once or twice a week cardiotocography (CTG) and ultrasound surveillance in secondary care from 41 weeks onwards and labour induction at 42 weeks, or labour induction starting at 41 weeks [23]. Until now, the interdisciplinary agreement between the Royal Dutch Organisation of Midwives (KNOV) and the Dutch Society for Obstetrics and Gynaecology (NVOG) concerning post-term pregnancy in the Netherlands, indicates secondary care and labour induction from 42 weeks onwards [8, 24]. However, more and more hospitals are converting their policy to induction of labour at 41 weeks, though there is no consensus concerning this change of policy. Opponents argue that hospitalised labour will diminish physiological birth, with increased rates of pain treatment, and operative delivery (vacuum/forceps c.q. Caesarean section) resulting in more negative birth experiences and an increase in costs with doubtful perinatal benefits [20, 25–28]. Unfortunately, data reflecting the Dutch situation, comparing induction of labour at 41 weeks with expectant management until 42 weeks, are lacking.

Observational data of the Dutch Perinatal Registry (PRN 2000 - 2006) show very small, but increasing rates of adverse perinatal outcomes such as Apgar score <7 (40 + 0-40 + 6 weeks 0.9%, 41 + 0-41 + 6 weeks 1.1% and ≥ 42 weeks 1.4%), meconium aspiration syndrome (40 + 0-40 + 6 weeks 0.12%, 41 + 0-41 + 6 weeks 0.21% and ≥ 42 weeks 0.25%) and NICU admission (40 + 0-40 + 6 weeks 0.49%, 41 + 0-41 + 6 weeks 0.62% and ≥ 42 weeks 0.91%), among births from singleton pregnancies [29].

However, perinatal mortality (up to 28 days) of singletons born between 41 + 0 – 41 + 6 weeks (0.16%) was comparable to perinatal mortality between 40 + 0 – 40 + 6 weeks (0.13%) and between 39 + 0 – 39 + 6 weeks (0.16%) [30]. PRN data also showed an increase in operative vaginal delivery when labour is induced beyond 41 weeks compared to induction at term (37 + 0-39 + 6 9.8%, 40 + 0-40 + 6 weeks 12.4%, 41 + 0-41 + 6 weeks 14.6% and ≥ 42 weeks 17.1%) [31].

Previous studies comparing expectant management and induction of labour in high risk pregnancies showed a discrepancy between observational PRN data and data from randomised trials (Digitat trial (growth retardation) and Hypitat trial (hypertension at term)) [32, 33]. The PRN database indicated an increased risk of operative vaginal delivery after induction of labour, whereas the subsequent randomised clinical trials showed that such an effect was absent [32–34]. Also PRN data showed a significant increase in Caesarean section after labour induction at or beyond 41 completed weeks compared to spontaneous onset of labour. However, Caesarean section rates in the Netherlands between 2000-2006 are much lower than in many other western countries (for singletons overall 9.4% between 41 + 0-41 + 6 weeks and 16.6% beyond 42 weeks) thus hampering the extrapolation of results from international studies, and emphasising the importance of this trial [29, 30].

Ethnic differences are likely to play an important role in post-term pregnancy. The mean duration of pregnancy is shorter in women from African origin and Indian origin as compared to Caucasian women [35]. Indeed, the incidence of stillbirth is higher from 41 weeks onwards among women from African and Surinam-Hindustan (Indian) origin as compared to Caucasian women [35, 36]. Though guidelines are not adjusted yet to ethnic origin, we will register ethnicity in our study.

We are not aware of other ongoing studies that are similar to the present study proposal or related to the problem discussed here (national or international). The issue of the timing of induction of labour in post-term pregnancy has been addressed in many studies showing that labour induction should be offered in case of post-term pregnancy because of the increased risk of perinatal mortality and morbidity [8, 19].

The 41-42 weeks dilemma considers a large proportion of pregnant women, as with a policy of labour induction at 41 weeks 18% (31,166/173,099) of all pregnant women in The Netherlands would be induced, compared to 1.5% (2,525/173,099) at 42 weeks [37]. With a policy of expectant management 68.7% will have spontaneous onset of labour between 41 + 0 – 41 + 6 weeks [37]. Before the introduction of the most recent guideline on management of post-term pregnancy in 2007 [38], there were less inductions of labour between 41 + 0 and 41 + 6 weeks (2006 18,2% (30,903), 2007 18,0% (30,151) and more deliveries after 42 weeks (2006 4,9% (8,312), 2007 4,5% (7,550)) [39].

Because of the controversy on this issue between caregivers, and the fact that the policy in surrounding countries is different, we feel that a nationwide randomised clinical trial is the obvious and necessary step to come to a multidisciplinary guideline regarding (impending) post-term pregnancy. We will conduct a randomised controlled trial to evaluate the effectiveness of a policy of labour induction at 41 weeks compared to expectant management until 42 weeks in women without contra-indications for expectant management.

## Methods/design

The study is set in the Dutch Obstetric Consortium: a collaboration of obstetric centers in the Netherlands in cooperation with the Midwifery Research Network of the Netherlands (MRNN) [40]. Approximately 200 centers, including university hospitals, teaching hospitals, non-teaching hospitals and midwifery practices will participate in this trial [24].

We will ask obstetrical low risk women ≥ 18 years with a singleton pregnancy in stable cephalic position and a certain gestational age of 40 + 5 - 41 + 0, based on first trimester ultrasound and without contra-indications for expectant management until 42 weeks for consent to participate in our study and to be allocated to induction of labour at 41 + 0/+1 weeks or at 42 + 0 weeks. Exclusion criteria are age <18 years, uncertain gestational age, obstetrical indications for secondary care (e.g. hypertension (systolic 140 mmHg and/or diastolic 90 mmHg or more), proteinuria (≥3 g/L), pre-existent maternal heart or kidney diseases, gestational diabetes, previous Caesarean section, multiple pregnancy, intra-uterine growth retardation) and non-reassuring fetal status (no fetal movements, abnormal fetal heart rate, known fetal abnormalities which could influence perinatal outcome, including abnormal karyotype, ruptured membranes at time of randomisation and a non-reassuring fetal status at time of randomisation). The results of the randomised clinical trial will be analysed according to the intention to treat principle.

### Intervention group: induction of labour at 41 + 0 or 41 + 1 weeks

Women randomised to induction of labour will be referred to the cooperating hospital for induction of labour according to local protocol. Induction of labour will be started at 41 + 0 to 41 + 1 weeks. Women with a cervix that is judged to be ‘ripe’ at vaginal examination (Bishop Score of 6 or more), will have labour induced with amniotomy followed by intravenous oxytocin according to local protocol. In case rupturing of membranes is not possible, cervical ripening will be accomplished in accordance with our national guidelines. In case the cervix is judged to be still unripe the day after priming, cervical ripening will be repeated. All patients in the intervention group will be monitored until after delivery.

### Control group: expectant management until 42 weeks

Women allocated to expectant management await spontaneous onset of labour until 42 weeks. If labour has not started, monitoring is according to local protocol. This reflects current care in The Netherlands. Monitoring can consist of consultations, electronic fetal heart rate monitoring and ultrasound assessment of amniotic fluid. An increase of the frequency of these checks as well as admission to the hospital is based on the judgment of the midwife or clinician in charge as usual. In the expectant management group, intervention will occur in case the fetal condition does not justify expectant management, such as reduced fetal movements reported by the mother, non-optimal fetal heart rate on CTG or oligohydramnios [12]. If an indication for induction of labour occurs, such as prelabour rupture of membranes for >24 hours or meconium stained amniotic fluid, referral to secondary care for labour induction is indicated according to the management strategies which are recorded in the national Obstetrical Indication List [41]. All diagnostic tests and interventions between randomisation and birth are registered in the case report form. Protocol violation is noted in the case report form with the reason of switch of policy to induction of labour. Women with uncomplicated pregnancies who are still in primary care will be referred to secondary care at 42 + 0 weeks for induction of labour following the procedure as stated for the intervention group.

### Outcome measures

#### Primary

Primary outcome will be a composite of perinatal mortality and neonatal morbidity. Adverse perinatal outcome is defined as a composite of perinatal mortality, a 5-minute Apgar-score below 7 and/or an arterial pH below 7.05 (as in other Consortium studies [32, 33]), meconium aspiration syndrome, plexus brachialis injury (with and without association with shoulder dystocia (additional manoeuvres to deliver shoulders)) and/or NICU admission (level of care and duration). Meconium aspiration syndrome is defined as respiratory distress in the first four hours after birth in presence of meconium stained amniotic fluid and categorised as severe (requiring assisted mechanical ventilation) or moderate (requiring oxygen for at least 48 hours or at a concentration of 40 percent or greater but without mechanical ventilation).

#### Secondary

Secondary outcomes will be maternal outcomes such as operative delivery (operative vaginal delivery, Caesarean section), need for analgesia (epidural, remifentanil, pethidin), post-partum haemorrhage ≥ 1000 ml and severe perineal injury (third- or fourth-degree perineal tear).

Maternal preferences, satisfaction, wellbeing and anxiety will be assessed alongside the trial [42, 43].

### Measurements

When a patient fulfils the study criteria and written informed consent is obtained, clinical data such as age, height, weight before pregnancy, ethnicity, highest finished education and social economic status based on postal code are collected at study-entry [44]. Obstetric history and level of care in current pregnancy are registered. Cervical ripeness will be assessed by digital examination of the cervix. The acquired Bishop score (based on dilatation, effacement, consistency, position and engagement) will be noted and fetal condition will be checked according to local protocol. Eligible women will be randomised subsequently.

After randomisation, number and lengths of admissions is noted. In the expectant management group, level of care and number of (outpatient clinic) visits is reported. At each visit, maternal and fetal assessments are recorded.

At the onset of labour all relevant data will be collected including start of labour. Data on first, second and third stage of labour are collected, including treatment for pain relief, mode of delivery and adverse perinatal and maternal outcomes.

Perinatal and maternal mortality and morbidity will be specified.

Data will be collected until women and child are discharged home for the first time or when deceased. If a participant withdraws from the study, the reason (lost to follow up, withdrawal of consent, prematurely stop of study) will be registered.

We will use standardised case report forms that have been established in previous studies.

Apart from the collection of clinical data, a sub-cohort of women will complete questionnaires addressing health related quality of life and wellbeing (EQ 6D [45], state anxiety (STAI [46]), preferences and satisfaction (SSQ [47, 48], LADY-X [49]), as well as questionnaires containing information on pain (PCS [50], NPRS/VAS) [51, 52]. Questionnaires will be completed at baseline after randomisation, and 6 weeks after delivery. Women can fill out the questionnaires online or on paper.

Women who do not give consent for randomisation will be treated according to the local protocol and they will be followed in a prospective cohort study.

### Follow up of women and infants

The last questionnaires will be filled out at 6 weeks post-partum. Informed consent will be asked for future follow up studies.

### Procedures, recruitment, randomisation and collection of baseline data

Randomisation will be performed through ALEA, a web-based software program for randomisation in clinical trials. The database is located in the central data collection unit in the Academic Medical Center in Amsterdam. Randomisation procedure is by individual randomisation. Women will be randomly allocated to either induction of labour or expectant management. We will collect data from women who refuse randomisation due to a strong preference for one of the treatment options or because they want to follow local policy. The study will be an open label study, as it is impossible to blind the health care workers and patients for the strategy to which the woman is allocated.

### Data safety

This nationwide trial will be carried out by midwives (primary care) and gynaecologists (secondary care). To ensure the quality of this study and to minimize the protocol violations, a website is launched with all study information. All sites will be informed by the researchers on the procedures. All sites have the possibility to consult (in person or by phone) the researchers of the study group and an email address is available for non urgent questions. All data are entered by research midwives or research nurses or trained medical students. After 900 inclusions, an interim analysis will be performed on safety. The operating procedures of the study are discussed with a data safety monitoring board, an independent group of experts who gave their approval to the design of the study safety.

### Serious adverse events

The following serious adverse events (SAE) will be identified: Perinatal death, maternal death, severe neonatal morbidity (NICU admission), severe maternal morbidity (IC/CCU admission), event related to induction of labour and uterine rupture, asphyxia and meconium aspiration with admission of the neonate on the NICU/High Care department. The Data Safety Monitoring Board will be informed if three SAE of the neonate will occur or one maternal SAE. All SAE are reported to the main investigators within 24 hours. They will report to the data safety monitoring board.

### Ethical consideration and trial registration

This study has been approved by the national central committee on research involving Human Subjects (CCMO- NL 38455.018.11), by the Medical Ethical Committee, Academic Medical Center, Amsterdam the Netherlands (METC: 2011/361). The study will be a multicenter randomised controlled trial. The participating hospitals got approval of their local boards. The trial was registered at the Dutch Trial Register (Nederlands Trial Register): NTR3431.

### Statistical issues

#### Sample size calculation

Sample size was calculated for non-inferiority testing using software Queary Advisor 7.0. Based on the incidence of the composite adverse perinatal outcome, the sample size is calculated at 900 women per group (1800 women in total) [53]. With this sample size, a two-group large-sample normal approximation test of proportions with a one-sided 0.050 significance level will have 80% power to reject the null hypothesis that labour induction and expectant monitoring are not equivalent (the difference in proportions, is 0.020 or further from zero in the same direction) in favour of the alternative hypothesis that the proportions in the two groups are equivalent, assuming that the expected difference in proportions on composite adverse perinatal outcome is 0.000 and the proportion in the standard group is 0.030.

When there is no equivalence this sample size (900 patients per group) will allow us to have 85% statistical power to detect 2% reduction in the risk of composite perinatal mortality and neonatal morbidity from 3% to 1%.

#### Data analysis

The results of the randomised clinical trial will be analysed according to the intention to treat principle. The effectiveness of labour induction at 41 weeks versus expectant management until 42 weeks will be assessed by calculating relative risks and 95% confidence intervals. Time to delivery will be compared using Kaplan-Meier curves and log-rank tests.

## Discussion

The policy regarding the timing of labour induction for (impending) post-term pregnancy is still under debate because of the inconclusiveness of the literature whether or not labour should be induced at 41 weeks or at 42 weeks for the prevention of adverse perinatal outcome. Most studies on labour induction or expectant management for (impending) post-term pregnancy started intervention beyond 41 weeks and continued expectant management far beyond 42 weeks. Until now, the Dutch guideline on post-term pregnancy indicates labour induction at 42 weeks. However, policy is moving towards labour induction at 41 weeks, though there is no consensus on this policy. This study will provide sufficiently precise and unbiased evidence on the difference between both strategies in perinatal and maternal outcome and patient preferences.

When our study shows that the incidence of poor neonatal and maternal outcome is very low and comparable with both strategies, this will in itself be an argument against intervention. Maternal preferences will then be leading in the choice between induction or expectant management. This study will help to achieve an evidence-based management strategy concerning impending post-term pregnancy.

We will adhere to the CONSORT guidelines for reporting the trial.
